# Pervasive, Preferential Flow through Mega‐Thick Unsaturated Zones in the Southern Great Basin

**DOI:** 10.1111/gwat.13187

**Published:** 2022-03-09

**Authors:** Tracie R. Jackson, Joseph M. Fenelon, Seth R. Gainey

**Affiliations:** ^1^ U.S. Geological Survey Nevada Water Science Center 500 Date St, Boulder City NV 89005 USA

## Abstract

Recharge from preferential flow through mega‐thick (100–1000 m) unsaturated zones is a pervasive phenomenon, as demonstrated with a case study of volcanic highland recharge areas in the Great Basin province in southern Nevada, USA. Statistically significant rising water‐level trends occur for most study‐area wells and resulted from a relatively wet period (1969–2005) in south‐central Nevada. Wet and dry winters control water‐level trends, with water levels rising within a few months to a year following a wet‐winter recharge event and declining during sustained dry periods. Even though a megadrought has persisted since 2000, this drought condition did not preclude major recharge events. Modern groundwater reaching the water table is consistent with previous geochemical studies of the study area that indicate mixing of modern and late Pleistocene recharge water. First‐order approximations and simple mixing models of modern and late Pleistocene water indicate that 10% to 40% of recharge is preferential flow and that modern recharge may play a larger role in the water budget than previously thought.

## Abstract 2

*Article impact statement*: Recharge from preferential flow is common in fractured rocks with mega‐thick unsaturated zones in the southern Great Basin.

## Introduction

Net infiltration of water through the unsaturated zone is driven by two hydrodynamic processes: diffuse and preferential flow (Nimmo 2005, 2020a, 2020b; Nimmo et al. 2021). Diffuse flow is the slow infiltration of water through the unsaturated zone, primarily through microscopic pore spaces, and is dependent on bulk medium properties (Nimmo 2020a). Preferential flow is the fast movement of water through a small fraction of the unsaturated zone and occurs because of high water content and (or) interconnected macropores or fractures (Nimmo 2021; Nimmo et al. 2021).

The travel time for water to move through the unsaturated zone to the water table is dependent on unsaturated‐zone thickness, effective porosity, and hydrodynamics (Flint et al. 2004; Nimmo et al. 2005). Travel times are short (less than 1 year) in areas with thin unsaturated zones (less than 5 m) (Delin et al. 2000). However, travel times in areas with thick (10–100 m) to “mega‐thick” (greater than 100 m) unsaturated zones can be short (months to years) or long (centuries to millennia), depending on effective porosities and whether preferential flow occurs (Fabryka‐Martin et al. 1998; Nimmo 2021). For example, travel times will be faster for preferential flow through fractured rock with low‐effective porosity, compared to diffuse flow through an alluvial matrix with high‐effective porosity (Nimmo 2005).

Preferential flow through thick to mega‐thick unsaturated zones in arid and semi‐arid regions is not uncommon (Scanlon et al. 1997; Guerin 2001; Nimmo 2021; Nimmo et al. 2021) and has been documented in North America (Davidson et al. 1998; Flint et al. 2002; Izbicki et al. 2002; Nimmo et al. 2002; Maxwell 2010), Asia (Li et al. 2017; Huang et al. 2019), the Middle East (Nativ et al. 1995), and Africa (Butler and Verhagen 2001). The lithologies of these unsaturated zones are diverse, composed of fractured basalts (Nimmo et al. 2002) and tuffs (Davidson et al. 1998; Ebel and Nimmo 2013), glacial sedimentary deposits (Li et al. 2017; Huang et al. 2019), carbonates (Butler and Verhagen 2001), alluvium (Izbicki et al. 2002), and other lithified and unlithified sedimentary materials (Butler and Verhagen 2001). These studies demonstrate that preferential flow of water through the unsaturated zone occurs over a wide range of geographic areas, diverse lithologies, and over a substantial range of thicknesses.

This paper demonstrates that recharge from preferential flow through mega‐thick unsaturated zones is a pervasive phenomenon in the southern Great Basin, using a case study of volcanic highland recharge areas in southern Nevada. Precipitation and water‐level data are used to show that recharge from wet winters causes corresponding water‐level rises in wells within 3 to 12 months following recharge events. A conceptual model is developed to explain rising water‐level trends from recharge in highland areas, where unsaturated zones are between 100 and 1300‐m thick. Results of this work demonstrate that mega‐thick unsaturated zones do not preclude modern recharge to groundwater systems and that observed rising water‐level trends are driven by preferential flow.

### Description of Study Area

**Figure 1:**
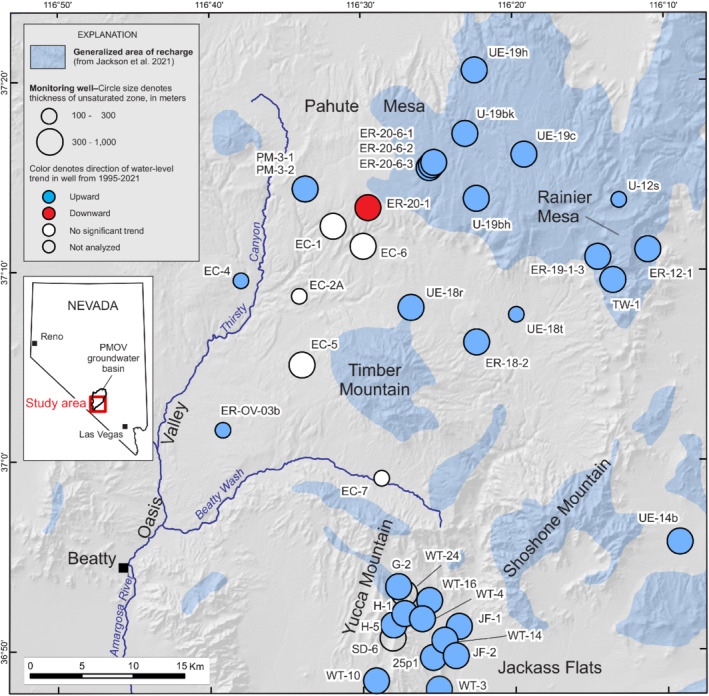
Physiography and location of wells in the study area. Study area is part of the Pahute Mesa–Oasis Valley (PMOV) groundwater basin (Jackson et al. 2021).

The study area comprises volcanic highland areas of Pahute Mesa, Rainier Mesa, Timber Mountain, Yucca Mountain, and Shoshone Mountain (Figure 1), which occur in the Great Basin province in southern Nevada (Harrill and Prudic 1998). These highland areas have land‐surface altitudes between 1500 and 2350 m above sea level and unsaturated‐zone thicknesses between 100 and 1300 m.

The volcanic highlands of the study area occur within and adjacent to a set of at least seven Miocene nested calderas (Sawyer et al. 1994; Grauch et al. 1999). Eruptions from these calderas deposited from 750 to as much as 4000 m of ash‐fall and ash‐flow tuffs, lavas, and breccia flows (Blankennagel and Weir Jr. 1973; Sawyer et al. 1994; U.S. Department of Energy 2020). Underlying the volcanic deposits are Paleozoic and Proterozoic carbonate and siliciclastic rocks. Basin and Range extensional forces have caused fracturing and normal faulting of the rocks, providing the primary pathways for flow of water through the unsaturated and saturated zones (Winograd and Thordarson 1975).

The climate of the study area is arid to semi‐arid, characteristic of a high desert region. Average summertime maximum temperatures range from 30 °C to 33 °C, and average wintertime minimum temperatures range from −2 °C to 7 °C (NOAA 2021a). Average annual precipitation in the highland areas of the study area ranges from about 19 cm on Pahute Mesa and Yucca Mountain to about 30 cm on Rainier Mesa (NOAA 2021a).

In southern Nevada, most recharge is derived from precipitation (rain or snow) in highland areas that occurs during the winter season (Winograd et al. 1998). The winter season is defined as the period from October 1 to March 31. Recharge is limited or nonexistent during summer because high temperatures and growing plants drive the process of evapotranspiration, which consumes nearly all infiltration (Smith et al. 2017).

## Methods

Groundwater‐level and precipitation data were compiled to analyze water‐level trends and develop a conceptual model to explain rising water‐level trends. For clarity, well names are italicized in this paper.

### Compiled Data

Water‐level data were retrieved from USGS (2021). Compiled water‐level data are periodic (mostly quarterly) measurements at 38 wells within the study area (Table S1). Selected wells have long‐term (20+ year) water‐level records with water‐level measurements between 1995 and 2021. Water levels in these wells were analyzed previously (Jackson and Fenelon 2018; Elliott and Fenelon 2021), and only levels previously interpreted to represent natural hydrologic conditions in the groundwater system were used in this analysis. Water levels affected by groundwater pumping, nuclear testing, or other anthropogenic stresses were filtered from datasets.

Daily precipitation data were compiled from precipitation stations in Pahute Mesa, Rainier Mesa, and Beatty, Nevada. Precipitation records from individual stations in these areas were stitched together to construct long‐term precipitation records, as outlined in the Supporting Information (Table S2). Beatty precipitation was used as a proxy for Yucca Mountain precipitation because Beatty is relatively close (20 km away; Figure 1) and Yucca Mountain does not have a precipitation station with a long‐term record. In addition, a water‐level trend analysis indicates that Beatty precipitation is the most representative precipitation station for recharge patterns in the Yucca, Timber, and Shoshone Mountain areas (Jackson and Fenelon 2018). Precipitation data were retrieved from NOAA (2021a), WRCC (2021a) and CEMP (2021).

A century‐scale (1900–2021) precipitation record was retrieved from WRCC (2021b). The century‐scale precipitation record is a composite of monthly precipitation data compiled from individual stations in the south‐central Nevada climate division (WRCC 2021b). The century‐scale precipitation record is referred to as the south‐central Nevada precipitation record.

### Statistical Analysis of Water‐Level Trends

Statistical methods were used to assess whether water levels in wells with unsaturated zone thicknesses of greater than 100 m have an upward, downward, or no significant trend for the period 1995 to 2021. This period was selected to provide a consistent record for analyzing water‐level data. Water‐level data were averaged by water year (October 1–September 30) from 38 wells (Table S1) and then analyzed statistically for trends using the Mann–Kendall trend test and Kendall's tau correlation coefficient (Mann 1945; Kendall 1975). Annually averaged water levels were used in the analysis so that periods of more frequent or sparse data collection did not bias results.

The Mann–Kendall trend test was used to assess the presence of a monotonic upward or downward water‐level trend at a well for the period 1995 to 2021. The Mann–Kendall trend test is a nonparametric method, which does not require water‐level data to be either normally distributed or to have a linear trend (Helsel et al. 2020). Kendall's tau correlation coefficient was computed to measure the strength of the monotonic trend in water‐level data. Kendall's tau values range between −1 and 1. A Kendall's tau value equal to 0 indicates no monotonic trend, which is the null hypothesis. A Kendall's tau equal to 1 indicates a strong rising trend, and a value equal to −1 indicates a strong declining trend. Rising and declining trends are considered highly significant if the level of significance (*p*‐value) is less than 0.001 and significant if the *p*‐value is less than 0.01.

### Estimation of Recharge Using Standardized Precipitation Index

Spatially varying recharge patterns in the study area were developed using a standardized precipitation index (SPI) of long‐term precipitation records. An SPI has values ranging from −3 to 3, where values represent the number of standard deviations by which precipitation deviates from the long‐term mean, assuming precipitation is normally distributed (McKee et al. 1993; Guttman 1999). Because precipitation is not normally distributed, the SPI is computed by transforming a long‐term precipitation record using a Gamma distribution and then fitting the transformed precipitation data to a normal distribution (Guttman 1999). The SPI was calculated using the methodology described in WMO (2012) and program files from NDMC (2021).

Potential recharge was estimated from the Rainier Mesa, Pahute Mesa, Beatty (Yucca Mountain area), and south‐central Nevada precipitation records. For each precipitation record, precipitation was summed during the winter months (October 1–March 31) and the 6‐month (winter) SPI computed (Table S3). Six‐month (winter) SPI values greater than 1 indicate wet climatic conditions (Guttman 1999). Therefore, in the study area, 6‐month (winter) SPI values greater than 1 are assumed to represent wet winters that contribute recharge to the groundwater system in areas with mega‐thick unsaturated zones (Jackson and Fenelon 2018).

Potential recharge was calculated as winter precipitation greater than a threshold. The threshold was defined as a 6‐month (winter) SPI equal to 1. For example, the computed, 6‐month (winter) SPI was 0.16 m for the south‐central Nevada precipitation record. Winter precipitation greater than 0.16 m represents potential recharge during wet winters, whereas winter precipitation less than 0.16 m is assumed to be evapotranspired. Precipitation thresholds have been used in previous studies as a proxy for recharge events (French et al. 1996; Jackson and Fenelon 2018).

### Water‐Level Modeling

Water‐level models (WLMs) simulated water‐level trends in the 38 study‐area wells. The WLMs were generated using SeriesSEE, a Microsoft Excel® add‐in (Halford et al. 2012). Any use of trade, firm, or product names (e.g., Microsoft Excel®) is for descriptive purposes only and does not imply endorsement by the U.S. government.

A WLM is an analytical model that fits a synthetic curve to measured water levels. The synthetic curve is the sum of one or more time‐series components that likely explain the aquifer stresses causing the water‐level trend. Time‐series components include (1) recharge responses simulated with Gamma transforms of the 6‐month (winter) SPIs, where values above a threshold of 1 only were used because they represent wet winters; and (2) natural aquifer drainage, which is assumed constant and was simulated as a linear decline. The assumption of constant natural aquifer drainage is discussed in the section “Aquifer Stresses: Discharge and Recharge.” WLMs for Rainier Mesa, Pahute Mesa, and Yucca Mountain area wells used SPI values computed from the Rainier Mesa, Pahute Mesa, and Beatty precipitation records, respectively.

The use of a Gamma distribution to transform precipitation (and recharge) to a water‐level response is a well‐established method (Markovic 1965; Eagleson 1978; Aksoy 2000; O'Reilly 2004). A Gamma distribution uses positive values only, which is ideal for simulating recharge, which is either zero or a positive value. The distribution accounts for the timing and magnitude of recharge with respect to unsaturated‐zone thickness, hydraulic connectivity of fractures, and distance from the recharge source to the well (O'Reilly 2004).

**Figure 2:**
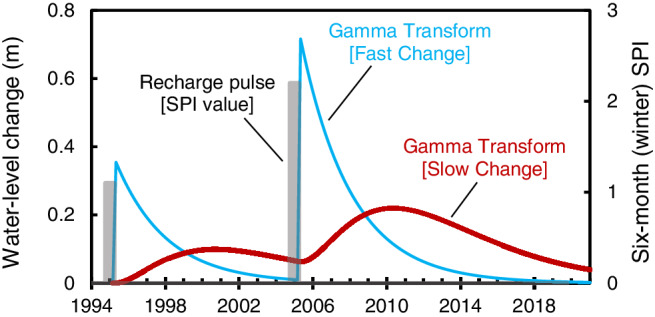
Water‐level responses from two conceptual recharge pulses using a Gamma transform.

A Gamma transform is the summation of individual Gamma distributions computed with time (Figure 2). Six‐month (winter) SPI values greater than 1 are a proxy for recharge and these values are the input variable for a Gamma distribution. One or two summed Gamma transforms were used to simulate the timing and magnitude of recharge (Figure 2).

The second component of the WLM, natural aquifer drainage, is assumed to occur at a constant rate equal to the long‐term recharge rate. Aquifer drainage was computed as total recharge divided by the estimation period. Total recharge was estimated by summing total winter precipitation, in meters, exceeding an SPI of 1 during the estimation period. An SPI of 1 is defined as one standard deviation above long‐term mean winter (October 1–March 31) precipitation from Rainier Mesa (1960–2020), Pahute Mesa (1964–2020), and Beatty (1973–2020) precipitation records. An SPI of 1 corresponds to values of 0.30, 0.15, and 0.14 m of precipitation for the Rainier Mesa, Pahute Mesa, and Beatty precipitation records, respectively. Winter precipitation that exceeds an SPI of 1 is assumed to recharge the groundwater system, and the sum of winter precipitation above an SPI of 1 is the total estimated recharge. Using Rainier Mesa as an example, total recharge was computed as 0.97 m from 1960 to 2020, which equates to an aquifer‐drainage rate of 0.016 m/year.

Synthetic water levels were fit to measured water levels by minimizing the root‐mean‐square error of differences between synthetic and measured water levels (Halford et al. 2012). Synthetic water levels are the sum of Gamma transforms representing recharge responses and a fixed linear line representing constant aquifer drainage. The amplitude, scale, and shape of the Gamma transform(s) were adjusted to match the synthetic curve to measured water levels using the software SeriesSEE (Halford et al. 2012).

### Mixing Models

Mixing models were used to estimate relative fractions of modern and paleo‐water recharging the groundwater system. Mixing models matched simulated and measured stable isotopes of oxygen (δ^18^O) compositions in study‐area wells. Simulated δ^18^O compositions were computed as relative fractions of modern and paleo‐water, using the following equation:

(1) $$\delta^{18}O_{\text{simulated}}=\left(f*\delta^{18}O_{\text{modern}}\right)+\left(\left[1-f\right]*\delta^{18}O_{\text{paleo}}\right)$$

where *f* is the fraction of modern water. The variables, δ^18^O_paleo_ and δ^18^O_modern_, are representative of paleo and modern δ^18^O compositions, respectively. These compositions were computed using a theoretical equation derived by Van der Straaten and Mook (1983, p. 60), which requires a recharge temperature to estimate δ^18^O compositions. A likely paleo‐recharge temperature is −5 °C for the study area (Benson and Klieforth 1989), whereas a modern recharge temperature of 4 °C was selected based on average, wintertime temperatures in highland areas of the study area (NOAA 2021a).

## Results and Discussion

Statistical analyses, WLMs, mixing models, and the development of a conceptual model were used to address the following questions:

1. How pervasive are rising water‐level trends in southern Nevada?
2. Can recharge from preferential flow explain the rising water‐level trends?
3. Why have water levels been rising within the last 25 years, despite drought conditions?
4. How can rising trends from preferential flow of modern recharge be reconciled with late Pleistocene groundwater?
5. What is the percentage of preferential recharge to the groundwater system?

### Rising Water‐Level Trends in Southern Nevada

Statistical analyses indicate that 32 of the 38 study‐area wells have significant upward water‐level trends from 1995 to 2021 (Figure 1; Table S4). Upward trends occur throughout the study area in wells that have unsaturated‐zone thicknesses ranging from 105 to 713 m and averaging 460 m. The few remaining wells with no significant trend (five wells) or a downward trend (one well) occur downgradient (southwest) of the Pahute Mesa recharge area.

The predominance of upward water‐level trends during the period of analysis suggests an overall wet period, where recharge exceeded discharge and groundwater storage increased. The absence of a water‐level trend in some downgradient wells indicates that either (1) recharge and discharge are in balance because water levels rose and declined over the 27‐year period of analysis; or (2) a well is isolated from the regional‐flow system by low‐permeability rocks and does not respond to annual or decadal recharge stresses.

### Stresses Affecting Rising Water‐Level Trends

Water‐level trends in the 38 study‐area wells were simulated using WLMs. WLMs were used to test the hypothesis that rising trends in highland areas with mega‐thick unsaturated zones can be explained by groundwater recharge from preferential flow.

**Figure 3:**
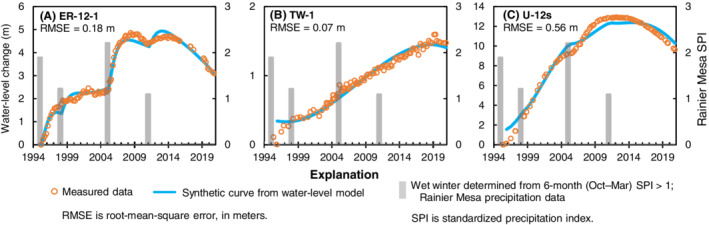
Comparison of synthetic to measured water levels from water‐level models at Rainier Mesa wells.

Comparisons of measured and synthetic water levels at Rainier Mesa wells demonstrate that water‐level trends can be explained when only recharge and aquifer drainage are simulated (Figure 3). In Rainier Mesa, water‐level responses to recharge are influenced by distance from the recharge source and the type of rock open to the well, rather than unsaturated‐zone thickness (Jackson and Fenelon 2018). Preferential recharge is conceptualized to occur through fractured carbonate rocks, which outcrop near well *ER‐12‐1* (Russell et al. 1996). Therefore, despite an unsaturated‐zone thickness of 464 m at well *ER‐12‐1*, water levels in the well have rapid and observable recharge responses to the 1995, 2005, and 2011 winters (Figure 3A). Recharge from the 1998 winter did not produce a strong measured water‐level response, but likely sustained the long rising trend that began in 1995. Water levels in well *TW‐1* have attenuated responses to recharge from the 1995, 1998, 2005, and 2011 winters because the well is downgradient of the recharge source and open to low‐transmissivity volcanic and carbonate rocks (Halford and Jackson 2020; Figure 3B). Well *U‐12s* is open to low‐transmissivity granitic rock, where low storage of disconnected fractures causes large observable recharge responses to the 1995 and 2005 winters (Figure 3C).

**Figure 4:**
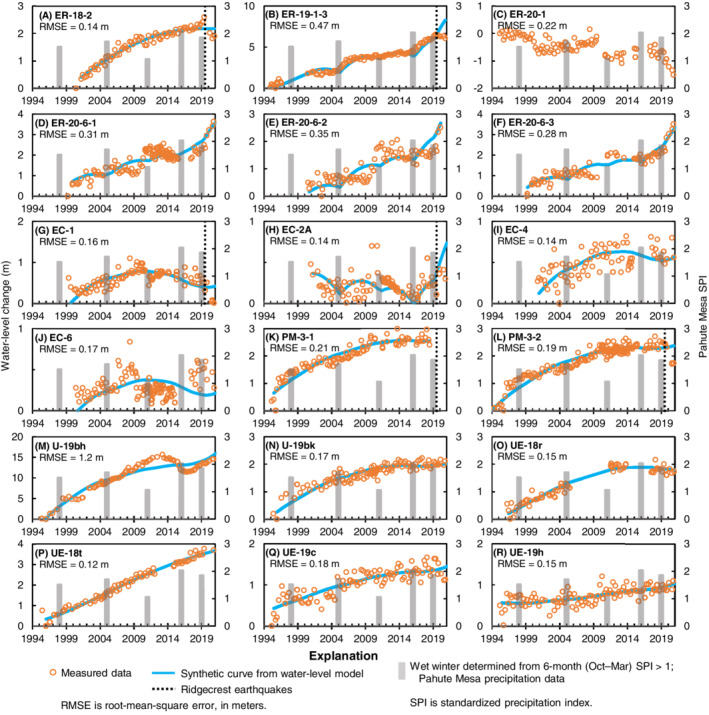
Comparison of synthetic to measured water levels from water‐level models at Pahute Mesa wells.

WLM results indicate that water‐level trends in Pahute Mesa wells are sustained by recharge from wet winters at Pahute Mesa. Measured and synthetic water levels have good fits when only recharge and aquifer drainage are simulated (Figure 4). Most of the long‐term rising trends do not show observable recharge responses to specific wet winters (Figure 4). However, the WLMs demonstrate that wells with sustained rising trends can be explained by recharge from the 1998, 2005, 2011, 2016, and 2019 winters. Measured short‐term water‐level declines in wells *ER‐18‐2*, *ER‐19‐1‐3*, *EC‐1*, *EC‐2A*, and *PM‐3‐2*, in response to the Ridgecrest earthquakes on July 4 and 6, 2019 (Elliott and Fenelon 2021; Wald and Collett 2021), were not simulated in the WLMs. Well *ER‐20‐1* has a declining trend from 1995 to 2021 because of anomalous downward shifts in water levels in 2000 and 2010, which were noted by Elliott and Fenelon (2021). These unexplained shifts cannot be modeled with a Gamma transform (Figure 4C). The likely pathway for preferential flow to Pahute Mesa wells, which have unsaturated‐zone thicknesses from 228 to 713 m, is through fractured and faulted volcanic rocks.

**Figure 5:**
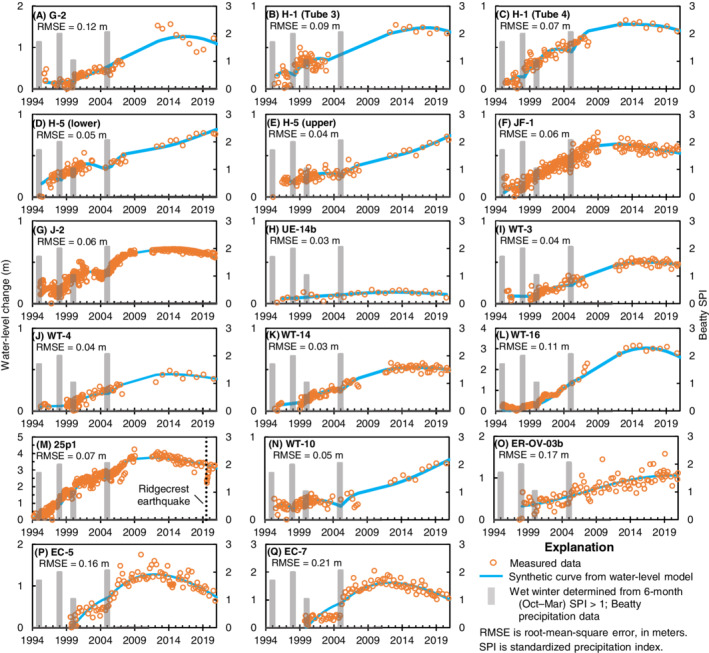
Comparison of synthetic to measured water levels from water‐level models at Yucca Mountain area wells.

Rising water‐level trends in Yucca Mountain area wells can be explained by recharge from wet winters. Measured and synthetic water levels have good fits when only recharge and aquifer drainage are simulated (Figure 5). Most wells have water levels with observable recharge responses to the 1995 and 2005 winters, and recharge from the 1998 and 2000 winters likely sustained the long rising trends that began in 1995 (Figure 5). In 13 of the 17 Yucca Mountain area wells, water‐level rises peaked around 2010 and have since been declining because of a 16‐year absence of wet winters in the southern part of the study area through 2021 (Figure 5). All wells analyzed in the Yucca Mountain area are open to volcanic rocks, except well *25p1*, which is open to carbonate rocks. Water levels in well *25p1* that abruptly declined in response to the Ridgecrest earthquakes (Elliott and Fenelon 2021; Wald and Collett 2021) were not simulated in the WLM. Unsaturated‐zone thicknesses range from 105 to 703 m at Yucca Mountain area wells, and the likely pathway for preferential flow to these wells is through fractured and faulted volcanic and carbonate rocks.

### Conceptual Model Explaining Rising Water‐Level Trends

A conceptual model was developed to explain why rising water‐level trends are expected in study‐area wells from 1995 to 2021. Based on a century‐scale (1900–2021) precipitation record (WRCC 2021b), the conceptual model represents a temporal recharge pattern for the study area and resulting hypothetical water‐level change. The conceptual model assumes that long‐term cumulative recharge and discharge are balanced with a resultant net change of zero in long‐term water levels and cumulative storage.

#### Steady‐State Assumption and Timescale

Observed rising water‐level trends reflect natural hydrologic conditions in the groundwater system (Figures 3, 4, 5) and are assumed to represent only part of a continuum of the steady‐state condition. Rising water levels contrast with the definition of steady state, where water levels do not change with time. However, the conceptual model assumes that water levels are in a state of dynamic equilibrium. Dynamic equilibrium recognizes that water levels are not stationary but fluctuate over the short‐term (years to decades) because of time‐varying natural stresses, such as recharge. Over a longer time (century) scale, steady‐state water levels are assumed to be unchanging.

Rising water‐level trends in the study area from 1995 to 2021 (Figure 1) indicate that the steady‐state timescale, where water levels remain constant, is greater than 27 years. The conceptual model described herein assumes a steady‐state timescale on the order of about a century. The timescale was tested using a precipitation dataset from 1900 to 2021 to determine if recent rising water‐level trends can be explained within the context of an approximate century‐scale steady‐state condition.

#### Aquifer Stresses: Discharge and Recharge

Naturally occurring hydrologic stresses affecting decadal water‐level trends in the study area are groundwater discharge and precipitation‐derived recharge. Groundwater discharge is synonymous with aquifer drainage previously described in this paper. Groundwater discharge in the study area occurs primarily from downgradient springs or seeps, which have nearly constant rates of annual discharge (Reiner et al. 2002; Halford and Jackson 2020). Aquifer discharge is constant with time because it is controlled by unchanging hydraulic properties of the groundwater system and a nearly constant regional hydraulic gradient. Naturally occurring water‐level changes minimally affect the gradient. For example, a 1‐m rise in water level at well *PM‐3‐2* would change the regional gradient by 0.003%. On the contrary, precipitation‐derived recharge varies temporally and spatially, and is the primary cause of decadal water‐level changes in wells.

The conceptual model uses the century‐scale (1900–2021), south‐central Nevada precipitation record (WRCC 2021b) to represent recharge patterns in the study area. Even though the century‐scale record does not equate to the true magnitude of precipitation in the recharge areas, the relative temporal distribution of dry and wet years is similar. Similarity is indicated by the high correlation of winter precipitation from Rainier Mesa, Pahute Mesa, and Beatty stations to winter precipitation from the south‐central Nevada precipitation record, with Spearman's rho correlation coefficients ranging from 0.79 to 0.90.

In the study area, most recharge is derived during winter months having greater‐than‐average precipitation, when evapotranspiration approaches zero (Winograd et al. 1998; Hershey et al. 2008). During most winters, when there is less‐than‐average to slightly above average precipitation, snowmelt infiltrating the root zone fills the soil‐moisture reservoir that was depleted by evapotranspiration during the previous summer (Smith et al. 2017). Little to no recharge occurs during these winters. Following winters with much greater‐than‐average precipitation (wet winters), snowmelt infiltrates the root zone, wetting the soil sufficiently to exceed its field capacity and allow excess infiltration to move downward as recharge (Smith et al. 2017). The conceptual model is simplified in that it does not consider soil moisture dynamics; specifically, the effect of annual changes in root‐zone water storage between wet and dry years.

**Figure 6:**
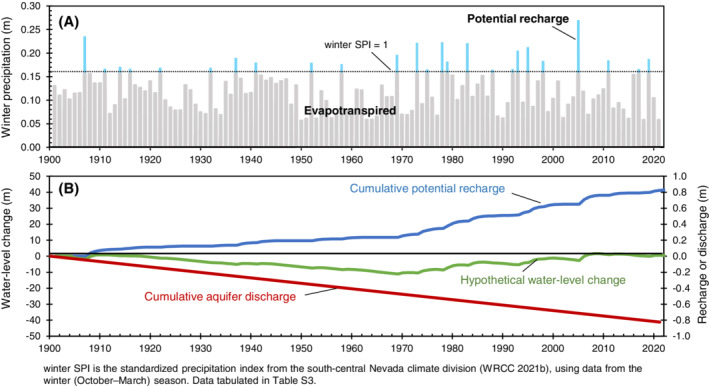
Steady‐state conceptual model for the study area, 1900–2021. (A) Winter precipitation distribution and potential recharge during wet winters. (B) Hypothetical water‐level change, cumulative potential recharge, and cumulative aquifer discharge.

Winter precipitation greater than 0.16 m represents potential recharge during wet winters, whereas winter precipitation less than 0.16 m is assumed to be evapotranspired (Figure 6A). Potential recharge does not indicate the absolute magnitude of recharge in the study area; rather, it represents the distribution of years when recharge occurred and the relative magnitudes. The recharge pattern in Figure 6A shows that the average annual recharge rate was three times greater after 1968 compared to the earlier part of the 20th century, indicating that the study area has been in a wet period from 1969 to 2021. The three‐fold increase in average annual recharge is associated with an increase in the percentage of wet winters from 15% between 1900 and 1968 to 28% between 1969 and 2021 (Figure 6A).

#### Hypothetical Water‐Level Record

A hypothetical, long‐term water‐level record was computed assuming steady‐state conditions over the 121‐year record; that is, cumulative recharge equals cumulative aquifer discharge. The hypothetical water‐level record is the sum of potential recharge and aquifer discharge (Figure 6B). Cumulative potential recharge with time is computed from summing winter precipitation greater than the winter SPI of 1 (Figure 6A). The aquifer‐discharge rate is assumed steady and was computed as total potential recharge from 1900 to 2021 divided by the 121‐year period. Potential recharge and aquifer discharge were translated to water‐level change by dividing values (in meters) by an assumed fractured‐rock effective porosity of 2% (Figure 6B; Winograd and Thordarson 1975).

**Figure 7:**
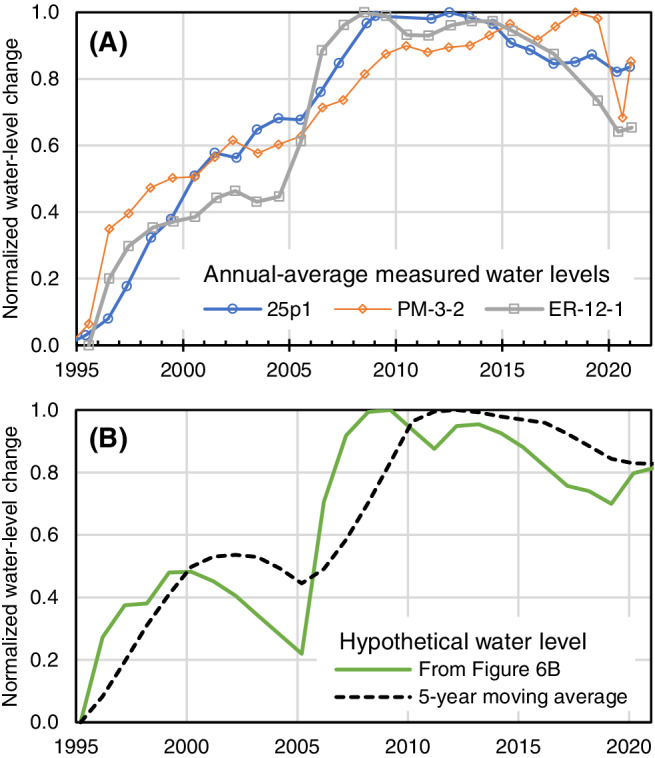
Comparison of (A) normalized, annually averaged, measured water‐level changes from three study‐area wells to (B) normalized, hypothetical water‐level change from Figure 6B.

The hypothetical water‐level record can explain the observed rising water‐level trends in the study area using an assumed century‐scale steady‐state period. Consistent with the concept of long‐term steady‐state conditions, the net hypothetical water‐level change for the period of record is zero (Figure 6B). However, the hypothetical water‐level change shows a declining trend from 1900 to 1968 and a rising trend from 1969 to 2021 (Figure 6B). If steady‐state conditions occur on a century timescale, measured water‐level trends in the study area from 1995 to 2021 will be upward, because the study area has been in a relatively wet period since 1969. The magnitude and exact pattern of water‐level trends will differ between the hypothetical water‐level record and water‐level records in study‐area wells because of differing rock porosities, recharge rates, and aquifer‐drainage rates; however, the general water‐level trends will be similar (Figure 7).

The conceptual model indicates that rising water‐level trends are the result of a relatively wet period since 1969. This result is consistent with a previous study, which indicates that rising water‐level trends have occurred since the 1960s in response to wet hydroclimatic conditions within the south‐central Nevada climate division (Dettinger and Schaefer 1995). A tree‐ring study of the southwestern United States also confirms that the latter part of the 20th century was exceptionally wet, with 1980 to 1998 being the wettest 19‐year period in the 1200‐year record (Williams et al. 2020). The extreme wet period was followed by a megadrought that has persisted since 2000 (Williams et al. 2020; NOAA 2021b). The drought condition does not preclude recharge events, however. For example, water‐level rises throughout south‐central Nevada from the 2005 wet winter demonstrate that highland recharge areas can receive a significant recharge event during a prolonged drought condition (Figures 3, 4, 5). Water‐level modeling results of this study suggest wet winters (seasonal events) are the primary driver of recharge, even during periods of drought. Measured water levels in wells (Figure 7A) show that water levels have begun to decline since 2010 in response to less frequent wet‐winter recharge events.

### Reconciling Modern Recharge with Late Pleistocene Groundwater

The conceptual model explaining steady‐state rising trends in the study area requires modern recharge to rapidly reach the water table. This rapid recharge must travel through mega‐thick unsaturated zones with depths between 100 and 1000 m. In apparent contradiction to the assumed occurrence of modern recharge in the study area are mean regional groundwater ages from the late Pleistocene. The validity of the conceptual model requires that modern recharge be reconciled with these old groundwater ages.

Numerous chemical and isotopic analyses have been done in the study area to characterize groundwater ages and flow paths (Benson and McKinley 1985; Thomas et al. 2002; Kwicklis et al. 2005; Rose et al. 2006; Hershey et al. 2008; Farnham et al. 2020). Stable isotopes of hydrogen (δD) and oxygen (δ^18^O) and radiocarbon ages between 10,000 and 20,000 years before present indicate that the *primary source* of regional groundwater in the study area is derived from the colder climatic period of the late Pleistocene (Benson and McKinley 1985; Farnham et al. 2020). However, these studies do not preclude input to the groundwater system of modern recharge, including preferential flow.

Chemical and isotopic studies of groundwater discharge from several deep tunnel complexes indicate modern recharge occurs in Rainier Mesa. The sub‐horizontal tunnels, constructed for underground nuclear testing, drain water near the water table and are greater than 300 m beneath Rainier Mesa (U.S. Department of Energy 2020). Clebsch Jr. (1961) measured bomb‐pulse tritium concentrations in E‐Tunnel discharge, and estimated groundwater ages between 0.8 and 6 years. Russell et al. (1987) reported that N‐Tunnel discharge responded rapidly to winter recharge events and used δD and δ^18^O to determine that the water is of recent meteoric origin. Norris et al. (1990) detected high concentrations of a bomb‐pulse chloride‐36 (^36^Cl) centered on a fault intersecting G‐Tunnel and attributed the ^36^Cl to preferential flow of modern recharge along the fault. Simple (Ebel and Nimmo 2013) and complex (Maxwell 2010) modeling of the unsaturated zone beneath Rainier Mesa also have indicated the possibility of preferential flow from modern recharge.

Elevated concentrations of bomb‐pulse tracers in groundwater indicate that modern recharge occurs by preferential flow through faults in Yucca Mountain. Elevated bomb‐pulse ^36^Cl concentrations have been measured in pore‐water samples from faults that intersect an 8‐km tunnel in Yucca Mountain, indicating fast preferential flow through 200–300 m of unsaturated rock within a 50‐year time frame (Liu et al. 1995; Fabryka‐Martin et al. 1998; Wolfsberg et al. 2000; Campbell et al. 2003). Preferential flow through the unsaturated zone in Yucca Mountain is localized to faulted areas, because bomb‐pulse ^36^Cl was not found at depth in areas unassociated with faults (Wolfsberg et al. 2000; Campbell et al. 2003). Stable isotopes (δD and δ^18^O), tritium, and ^14^C in pore waters from Yucca Mountain wells *WT‐24* and *SD‐6* indicate the presence of modern groundwater at multiple depths between 440 and 770 m (Figure 1; Yang 2000). Flint et al. (2002) concluded that Yucca Mountain groundwater is a mixture of modern and late Pleistocene groundwater based on measured Cl concentrations using a chloride‐mass‐balance method.

Pahute Mesa geochemical studies indicate an absence of modern recharge to the water table, based on stable isotopes and radiocarbon ages of groundwater (Kwicklis et al. 2005; Rose et al. 2006; Farnham et al. 2020). Farnham et al. (2020) concluded that the groundwater system beneath Pahute Mesa does not receive modern recharge, based on the observation of light stable isotope (δD and δ^18^O) concentrations. Groundwater ^14^C ages, which were corrected using δ^13^C and DIC, range between 10,000 and 18,500 years before present (Kwicklis et al. 2005; Farnham et al. 2020). Lack of a modern recharge signature likely results from the mixing of relatively small amounts of modern recharge water with a larger component of late Pleistocene recharge water, a topic discussed in the following sections.

### Estimated Fraction of Preferential Flow

Modern recharge water is attributed to preferential flow from wet winters. A first‐order approximation of the fraction of preferential flow entering the groundwater system was done using a scaling relation of measured water‐level data to the hypothetical water‐level record (see Appendix S1 in Supporting Information for analysis details). Based on this analysis, the fraction of preferential flow entering the groundwater system is estimated to be between 10% and 40%.

A preferential‐flow fraction of between 10% and 40% is consistent with simple δ^18^O mixing models. Mixing‐model results indicate that the relative fraction of modern water in Pahute Mesa groundwater samples generally ranges from about 10% to 20%, whereas modern‐water fractions in Yucca Mountain groundwater samples range from about 20% to 45% (see Table S5 in Supporting Information for analysis details). The authors recognize that the mixing‐model results presented in Table S5 are simplistic and ignore uncertainties associated with (1) modern and paleo recharge temperatures, which have ranges rather than single values; (2) unsaturated‐zone travel times, which vary depending on unsaturated‐zone thickness and geologic properties; and (3) analytical accuracy of δ^18^O compositions. Nevertheless, the purpose of the mixing‐model exercise was to demonstrate that a nonnegligible component of groundwater in the study area is modern. Mixing results should not be interpreted as exact fractions of modern and paleo‐water.

### The Paleo‐Recharge Phenomenon

The recharge conceptual model for the study area is that there are two recharge mechanisms: preferential flow from wet winters and diffuse paleo‐recharge. Paleo‐recharge is late‐Pleistocene precipitation that is percolating diffusely through pore spaces in the thick unsaturated zone to the water table (Kwicklis et al. 2005). Fenelon et al. (2016) estimated that 50,000,000 acre‐ft (62 km^3^) of predominantly paleo‐water is stored in the unsaturated zone beneath Pahute Mesa, and that the travel time for paleo‐recharge to percolate to the water table is on the order of 15,000 years.

Unsaturated‐zone processes control groundwater ages beneath Pahute Mesa, rather than saturated‐zone processes. This statement is substantiated by a groundwater‐flow model of the Pahute Mesa–Oasis Valley (PMOV) groundwater basin, which encompasses most of the study area (Fenelon et al. 2016). Groundwater‐flow paths in the PMOV basin are southwestward from Pahute Mesa through Thirsty Canyon and terminate at springs discharging in Oasis Valley (Figure 1; Fenelon et al. 2016). Computed travel times are less than 3000 years for nearly all flow paths from recharge areas to the Oasis Valley discharge area (Fenelon et al. 2016). Therefore, groundwater movement is relatively fast through the saturated system, compared to diffuse flow through the unsaturated zone. Late Pleistocene groundwater ages are expected beneath Pahute Mesa because groundwater ages are dominated by the large component of slow, steady paleo‐recharge to the water table.

**Figure 8:**
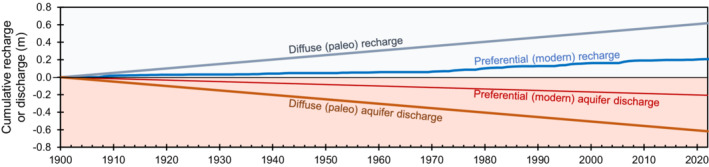
Steady‐state conceptual model for the study area, 1900–2021, showing cumulative recharge and aquifer‐discharge components attributed to 25% preferential (modern) and 75% diffuse (paleo) flow.

In the conceptual model of recharge and discharge (Figure 6A), potential recharge accounts for recharge from preferential flow (modern water source) and diffuse flow (paleo‐water). The estimated preferential‐flow fraction is between 10% and 40%, where 25% is the average of this range. Assuming that preferential and diffuse flow account for 25% and 75% of the potential recharge, respectively, a century‐scale, steady‐state water balance can be developed (Figure 8).

Because of the slow nature of diffuse infiltration, paleo‐recharge to the groundwater system is assumed to balance annually with an equal amount of aquifer discharge from the system (Figure 8). Paleo‐recharge is not observed in well hydrographs because the steady‐state nature of paleo‐recharge causes no net change in groundwater storage. Therefore, the hypothetical water‐level trend (Figure 6B) represents a water balance between preferential recharge and preferential aquifer discharge (Figure 8). For the case of preferential flow as a main driver of water‐level change, the hypothetical water‐level trend shown in Figure 6B is correct, but the magnitude of hypothetical water‐level changes is between 10% and 40% smaller than depicted in Figure 6B.

### Hydraulics of Modern Recharge

Rising water‐level trends indicate that recharge occurs throughout the study area. However, the conceptual model of recharge and discharge, which attributes rapid water‐level rises to wet winters, does not necessarily require modern recharge to occur uniformly across the water table. Water‐level rises can result from focused recharge along a fault in a rock unit with low‐effective porosity or beneath an ephemeral river channel. Focused recharge at the water table, analogous to a slug injection, will propagate outward to distant wells as a hydraulic‐pressure response. Hydraulic responses propagating more than a kilometer have been noted from large‐scale multiple‐well aquifer test investigations on Pahute Mesa (Garcia et al. 2017).

## Conclusions

Recharge from preferential flow through mega‐thick (100–1000 m) unsaturated zones likely is not limited to the southern Great Basin. Rapid water‐level responses to wet winters were observed in fractured volcanic, carbonate, and granitic rocks in the study area. The volcanic rocks consisted of interlayered bedded and welded tuffs and lava flows. The occurrence of preferential flow through a variety of thick rock sequences suggests that the phenomenon is independent of rock type. The more likely cause of pervasive preferential flow is the extensional tectonics of the Great Basin, which promote extensive fracturing and faulting. Consequently, any arid to semi‐arid area where rocks are extensively fractured and faulted may experience deep preferential flow.

The steady‐state, conceptual model can help predict how the groundwater system might respond to future climate change. Future climate in southern Nevada is expected to be warmer and drier, resulting in less recharge (Meixner et al. 2016), but with more extreme weather events (IPCC 2021). Less recharge will reset the century‐scale steady‐state regime, resulting in declining water levels as the preferential component of the system adjusts to the new normal. The larger component of the groundwater budget, diffuse flow, will take millennia to reset and will moderate century‐scale water‐level declines. More extreme weather events will lead to more extremely dry and wet winters. Extreme dry winters will deplete soil reservoirs, which likely will impact plant communities (less evapotranspiration). Contrarily, extreme wet winters may provide infrequent, but large pulses of preferential flow to the groundwater system, as was observed in the study area during the 2005 winter.

Results of this work demonstrate that modern recharge to groundwater systems can occur through mega‐thick unsaturated zones, and that modern recharge may play a larger role in the water budget than previously expected. For example, preferential flow of modern recharge may have implications for the ability of modern environmental tracers to reach deep water tables. The most likely pathways would be in areas of focused recharge beneath an ephemeral stream channel or along a fault. Therefore, evidence of modern‐day environmental tracers in a deep groundwater system should not be immediately discounted as sampling artifacts.

In groundwater systems with thick unsaturated zones, the infiltration rate below the root zone is not equivalent to the recharge rate at the water table. As demonstrated in this paper, shallow infiltration into the unsaturated zone is proportioned between diffuse and preferential components of recharge. The preferential component responds rapidly to modern‐day changes in precipitation and shallow infiltration, whereas the diffuse component can be lagged by thousands of years. The water budget presented in Figure 8 assumes that the diffuse component of shallow infiltration at land surface is equal to diffuse recharge at the water table. However, the diffuse component entering the unsaturated zone today is probably out of balance with the diffuse recharge entering the water table. Therefore, estimating recharge based on modern‐day infiltration must account for the time lag of the diffuse component, which may be out of sync with present‐day infiltration rates.

### Authors' Note

The authors do not have any conflicts of interest or financial disclosures to report.

## Supporting information


**Table S1**. Location and completion information for wells used in water‐level trend analysis.
**Table S2**. Location and altitude information for precipitation monitoring stations used to construct long‐term precipitation records. Table includes a description on the construction of long‐term precipitation records.
**Table S3**. Winter precipitation and standardized precipitation index values for Rainier Mesa, Pahute Mesa, Beatty, and South‐Central Nevada precipitation records.
**Table S4**. Analysis of water‐level trends, using the Mann–Kendall test, for study‐area wells
**Table S5**. Stable isotopic data (δ^18^O) and computed fractions of modern water for study‐area wells.
**Appendix S1**. Computation of the preferential‐flow fraction into the Pahute Mesa–Oasis Valley basin.
